# Supplementary material for: Use of large language model-based chatbots in managing the rehabilitation concerns and education needs of outpatient stroke survivors and caregivers
Source: Front Digit Health. 2024 May 9;6:1395501. doi: 10.3389/fdgth.2024.1395501 (PMC11111889; doi:10.3389/fdgth.2024.1395501)
Supplement: Supplementary Material S1 — Adapted questionnaire for patients and caregivers. [file Datasheet1.docx]

**Questionnaire**

1. What questions would you like to ask regarding your diagnosis or prognosis?
2. What questions would you like to ask regarding your physical symptoms and/or communication deficits?
3. What questions would you like to ask regarding changes in your physical capacity, if any, and their impact on your activities/roles/body image?
4. What questions would you like to ask regarding changes in your mood (such as view of self, view of the future, mental coping, and feelings of isolation)?
5. What questions would you like to ask regarding getting psychological support?
6. What questions would you like to ask regarding health information or support received, and/or communications with the hospital or your social environment?
7. What questions would you like to ask regarding the changes made in your engagement in activities (such as new ways of doing things, new roles, and new activities)?
8. What questions would you like to ask regarding the acceptance of your condition and/or adjustments that you have had to make since having a stroke?
9. What questions would you like to ask regarding social support?

1. What questions would you like to ask regarding your stroke rehabilitation program or process (such as nutrition, exercise, and access to resources or support groups)?

Please return this questionnaire to the clinic’s front desk, regardless of whether you completed it. Thank you for your support! If you are staying well, keep up the good job!
