# Supplementary material for: Use of large language model-based chatbots in managing the rehabilitation concerns and education needs of outpatient stroke survivors and caregivers
Source: Front Digit Health. 2024 May 9;6:1395501. doi: 10.3389/fdgth.2024.1395501 (PMC11111889; doi:10.3389/fdgth.2024.1395501)
Supplement: Supplementary Material S3 — Answers from chatbots. [file Datasheet3.docx]

**Supplementary Material 3 – Answers from Chatbots**

Date of queries: 5^th^ February 2024

Leading question: “I am a patient living with stroke in Singapore. I have a quick question about my stroke rehabilitation.”

| **S/N** | **Curated question** | **ChatGPT** | **Google Bard** |
| --- | --- | --- | --- |
| **1** | How long it will take for me to recover after my stroke? I've been putting in effort with exercises and medications, so is there a chance I can fully recover? | The duration and extent of stroke recovery vary greatly from person to person, and it's challenging to predict an exact timeline or guarantee full recovery. Factors such as the type and severity of the stroke, individual health, and the effectiveness of rehabilitation efforts all play crucial roles.  Recovery after a stroke is often a gradual process, and improvements can continue for months or even years. The first few months are typically considered the most critical for recovery, and many individuals experience significant gains during this time.  The goal of stroke rehabilitation is to maximize your functional abilities and quality of life. It's essential to continue with your exercises and medications as prescribed by your healthcare team. Consistency and dedication to rehabilitation efforts can contribute to better outcomes.  It's also crucial to maintain open communication with your healthcare professionals, as they can provide insights into your specific situation and adjust your rehabilitation plan accordingly. Additionally, support from family, friends, and support groups can play a significant role in the overall recovery process.  While some individuals may achieve near-full recovery, others may have residual challenges. The focus should be on optimizing your abilities and adapting to any lasting changes.  Always consult with your healthcare team for personalized advice and information regarding your specific case. They can provide the most accurate assessments based on your individual health and circumstances. | Unfortunately, I can't predict how long your stroke recovery will take or confirm your chances of a full recovery. Every stroke is unique, and the recovery process varies greatly depending on several factors, including:  * **Severity of the stroke:** More severe strokes generally have a longer and more challenging recovery compared to milder cases. * **Location of the stroke:** The specific area of the brain affected plays a significant role in determining the types of impairments and their potential for recovery. * **Your age and overall health:** Younger and healthier individuals often have a better chance of recovering more fully than older individuals or those with pre-existing health conditions. * **Timeliness of medical intervention:** Receiving prompt medical attention can significantly improve recovery outcomes. * **The intensity and adherence to your rehabilitation program:** Engaging actively in rehabilitation exercises and medication adherence are crucial for maximizing recovery potential.  While predicting the specific timeline is impossible, here's some general information:  * **Most significant improvements:** Studies suggest the **first 3-4 months** post-stroke see the most significant progress. * **Continued progress:** While slower, some improvements can continue well into the **first and second year**. * **Long-term outcomes:** Some individuals achieve full recovery, while others manage long-term or lifelong disabilities.  **Focus on maximizing your recovery:**  Instead of dwelling on unknowns, it's crucial to focus on the things within your control, like:  * **Actively participating in your rehabilitation program:** Attending all therapy sessions, diligently practicing exercises, and following your therapist's guidance are key. * **Maintaining a healthy lifestyle:** Eating a balanced diet, getting enough sleep, managing stress, and avoiding smoking can significantly impact your recovery journey. * **Staying positive and connected:** Maintaining a positive mindset and seeking support from loved ones or support groups can help you cope with challenges and stay motivated.  Remember, **consulting your doctor or therapist is essential** for personalized guidance and answers to your specific concerns about your recovery timeline and potential for full recovery. They can assess your individual case, consider all relevant factors, and provide you with the most accurate and relevant information. |
| **2** | How can I make sure I don't have another stroke? Are there things I should do or avoid doing to prevent it from happening again? | Preventing another stroke involves making lifestyle changes and managing risk factors. While I'm not a doctor, and it's important to consult with your healthcare team for personalized advice, here are some general recommendations to reduce the risk of recurrent strokes:  1. **Medication Adherence:** If your healthcare provider has prescribed medications for conditions like high blood pressure, high cholesterol, or diabetes, it's crucial to take them as directed. These medications help manage risk factors for stroke.  2. **Blood Pressure Control:** Maintain a healthy blood pressure through lifestyle modifications, such as a balanced diet, regular exercise, limiting alcohol intake, and taking prescribed medications if necessary.  3. **Manage Cholesterol Levels:** Follow a heart-healthy diet low in saturated and trans fats, exercise regularly, and take medications as prescribed to control cholesterol levels.  4. **Control Diabetes:** If you have diabetes, manage your blood sugar levels through a combination of a healthy diet, regular physical activity, and any prescribed medications or insulin.  5. **Quit Smoking:** If you smoke, quitting is one of the most effective ways to reduce your risk of stroke. Seek support from healthcare professionals or smoking cessation programs.  6. **Limit Alcohol Intake:** If you consume alcohol, do so in moderation. Excessive alcohol intake can contribute to high blood pressure and increase the risk of stroke.  7. **Maintain a Healthy Diet:** Eat a balanced and nutritious diet that includes plenty of fruits, vegetables, whole grains, lean proteins, and low-fat dairy products. This can help control weight, blood pressure, and cholesterol levels.  8. **Regular Exercise:** Engage in regular physical activity, as it can contribute to overall cardiovascular health. Consult with your healthcare team before starting any new exercise regimen.  9. **Weight Management:** Maintain a healthy weight through a combination of a balanced diet and regular exercise. This can help control various risk factors for stroke.  10. **Manage Stress:** Find healthy ways to cope with stress, such as relaxation techniques, mindfulness, or engaging in activities you enjoy.  11. **Regular Medical Check-ups:** Attend regular follow-up appointments with your healthcare provider to monitor your overall health and address any concerns promptly.  It's crucial to work closely with your healthcare team to create a personalized plan for stroke prevention based on your specific health needs and risk factors. They can provide guidance on lifestyle modifications and monitor your progress over time. | While I can't offer specific medical advice, I can share some general information about preventing a second stroke that might be helpful. However, it's crucial to remember that **consulting with your doctor or healthcare team is essential for creating a personalized prevention plan tailored to your specific situation and medical history.**   Here are some general steps you can take to reduce your risk of another stroke:  **Healthy lifestyle:**  * **Diet:** Adopt a healthy diet rich in fruits, vegetables, whole grains, and lean protein. Limit saturated and trans fats, cholesterol, and salt. Consider following a Mediterranean diet, which has been shown to be beneficial for heart and brain health. * **Exercise:** Engage in regular moderate-intensity physical activity for at least 30 minutes most days of the week. Even small changes like taking the stairs or going for a short walk can make a difference. * **Weight management:** Maintain a healthy weight. Being overweight or obese increases your risk of stroke and other health problems. * **Smoking cessation:** If you smoke, quitting is one of the most important things you can do to improve your health and reduce your stroke risk. * **Alcohol moderation:** Limit alcohol consumption. Excessive alcohol intake can raise blood pressure and increase your stroke risk. * **Stress management:** Find healthy ways to manage stress, such as yoga, meditation, or spending time in nature.  **Managing medical conditions:**  * **Blood pressure control:** High blood pressure is a major risk factor for stroke. Work with your doctor to monitor and control your blood pressure through medication and lifestyle changes. * **Cholesterol management:** High cholesterol levels can contribute to stroke risk. Follow your doctor's recommendations for managing your cholesterol levels through diet, medication, or other lifestyle changes. * **Diabetes management:** If you have diabetes, it's crucial to manage your blood sugar levels effectively to reduce your stroke risk. * **Sleep:** Aim for 7-8 hours of quality sleep each night. Poor sleep can contribute to high blood pressure and other health problems that increase stroke risk.  **Preventive medications:**  * Your doctor may prescribe medications like aspirin or blood thinners to help prevent blood clots that can lead to stroke, depending on your specific risk factors.  **Regular checkups:**  * Schedule regular checkups with your doctor to monitor your health and adjust your prevention plan as needed.  Remember, this is just general information, and it's crucial to **discuss your specific situation and questions with your healthcare team**. They can create a personalized plan that addresses your individual risks and needs to help you prevent another stroke and live a healthy life. |
| **3** | How do I get better after my stroke? What treatments and therapies are available locally, and is there anything new or special that can help me to recover faster? | Stroke recovery is a complex and individualized process, and the specific treatments and therapies you receive may depend on the type and severity of your stroke, as well as your overall health. It's crucial to work closely with your healthcare team to create a personalized rehabilitation plan. Here are some common components of stroke recovery:  1. **Physical Therapy:** Focuses on improving mobility, strength, and coordination. Exercises may target specific affected areas, helping you regain control and function.  2. **Occupational Therapy:** Aims to enhance your ability to perform daily activities, such as dressing, eating, and bathing. Occupational therapists work to improve fine motor skills and independence.  3. **Speech Therapy:** If you experience difficulty with speech, language, or swallowing, a speech therapist can provide exercises and strategies to improve communication and swallowing function.  4. **Medications:** Your healthcare provider may prescribe medications to manage risk factors, prevent further strokes, or address specific symptoms such as spasticity or pain.  5. **Rehabilitation Centers:** Inpatient or outpatient rehabilitation programs can offer comprehensive support and therapies, including physical, occupational, and speech therapy.  6. **Counseling and Psychological Support:** Dealing with the emotional impact of a stroke is an essential aspect of recovery. Counseling or support groups can provide emotional support and coping strategies.  7. **Assistive Devices:** Depending on your needs, your healthcare team may recommend assistive devices such as braces, walkers, or adaptive tools to aid in daily activities.  As for new or special interventions, ongoing research may lead to advancements in stroke rehabilitation. Experimental treatments, emerging technologies, or clinical trials could be considered, but these should be discussed with your healthcare team to determine their suitability for your specific case.  In Singapore, healthcare services are well-developed, and you should have access to a range of rehabilitation options. Public hospitals, private clinics, and rehabilitation centers may offer different programs and therapies.  Always consult with your healthcare team to discuss your progress, set realistic goals, and explore potential advancements in stroke recovery that may be suitable for your situation. They can provide guidance on the latest research and interventions that may benefit your rehabilitation process. | I understand that you have a question about your stroke rehabilitation in Singapore, but I am unable to provide medical advice. However, I can help you access resources and information that can assist you.   Here are some options:  **1. Consult your doctor or therapist:** They are the best people to answer questions specific to your condition and rehabilitation plan.   **2. Contact a support organization:** Several organizations in Singapore offer support and information for stroke survivors and their families. Here are a few:  * **Singapore National Stroke Association (SNSA):** [https://www.snsa.org.sg/](https://www.snsa.org.sg/) * **S3: Singapore's First Stroke-Focused Community Rehabilitation and Wellness Agency:** [https://www.s3.org.sg/](https://www.s3.org.sg/) * **Agency for Integrated Care (AIC):** [https://www.aic.sg/](https://www.aic.sg/)  **3. Use online resources:** The Ministry of Health Singapore provides information on stroke rehabilitation on their website: [https://www.healthhub.sg/a-z/medical-and-care-facilities/care-after-stroke](https://www.healthhub.sg/a-z/medical-and-care-facilities/care-after-stroke)  Remember, it's important to get your information from reliable sources and to consult with your medical professionals for any specific questions you have about your health. |
| **4** | How do I handle symptoms like hip and arm pain, stiffness, and swelling after my stroke? | I'm not a healthcare professional, but I can offer some general suggestions that may help. It's important to consult with your healthcare team for personalized advice. Here are some strategies that individuals recovering from a stroke may find useful for managing symptoms like hip and arm pain, stiffness, and swelling:  1. **Physical Therapy:** Work with a physical therapist to develop a tailored exercise program that focuses on improving mobility, strength, and flexibility. They can provide guidance on exercises specifically designed to address hip and arm issues.  2. **Medication:** Your healthcare provider may prescribe medications to help manage pain or reduce inflammation. It's important to take any prescribed medications as directed and to inform your healthcare team of any side effects.  3. **Hot and Cold Therapy:** Applying heat or cold to the affected areas may help alleviate pain and reduce swelling. Consult with your healthcare team to determine which approach is suitable for your situation.  4. **Compression Garments:** Compression sleeves or garments may be recommended by your healthcare professional to help reduce swelling in the affected arm. Ensure proper sizing and use as directed.  5. **Elevate the Arm or Leg:** Elevating the affected limb, especially when resting, can help reduce swelling. Consult with your healthcare team for guidance on the appropriate positioning.  6. **Gentle Range of Motion Exercises:** Perform gentle range of motion exercises to prevent stiffness and improve flexibility. These exercises should be done under the guidance of a healthcare professional or physical therapist.  7. **Manage Weight:** Maintaining a healthy weight can help alleviate stress on your joints and reduce the risk of exacerbating pain or swelling.  8. **Stay Hydrated:** Proper hydration is essential for overall health and can contribute to reducing swelling.  9. **Assistive Devices:** Depending on your specific needs, your healthcare team may recommend assistive devices, such as braces or splints, to support and stabilize your hip or arm.  Always consult with your healthcare team before starting any new exercise or treatment regimen, as they can provide guidance based on your individual health status and recovery process. If you experience persistent or worsening symptoms, it's crucial to seek prompt medical attention. | I understand you're experiencing hip and arm pain, stiffness, and swelling after your stroke, and I want to emphasize that I cannot provide medical advice. It's crucial to discuss these symptoms with your healthcare team or a qualified medical professional in Singapore. They can assess your specific situation, provide accurate advice, and recommend the best course of action for managing your symptoms effectively.  However, I can share some general information about these common concerns after stroke:  **Hip and arm pain:**  * **Possible causes:** Spasticity (muscle tightness), joint pain, nerve pain, or other post-stroke complications. * **Potential management options:** Medication (pain relievers, muscle relaxants), physical therapy, occupational therapy, splinting, assistive devices, and other interventions determined by your healthcare team.  **Stiffness:**  * **Possible causes:** Spasticity, decreased mobility, contractures (shortening of muscles). * **Potential management options:** Physical therapy, stretching exercises, splinting, heat therapy, and other interventions determined by your healthcare team.  **Swelling:**  * **Possible causes:** Lymphedema (fluid buildup), blood clots, or other medical conditions. * **Potential management options:** Elevation of affected limb, compression garments, massage therapy, and other interventions determined by your healthcare team.  **Remember:**  * **Early intervention is key:** Addressing these symptoms promptly can help prevent further complications and improve your recovery. * **Communication is crucial:** Don't hesitate to discuss any concerns or changes in your symptoms with your healthcare team. * **Support is available:** Seek support from rehabilitation specialists, support groups, and other resources dedicated to stroke recovery.  Here are some helpful resources in Singapore:  * **HealthHub:** [[https://www.healthhub.sg/a-z/medical-and-care-facilities/care-after-stroke](https://www.healthhub.sg/a-z/medical-and-care-facilities/care-after-stroke)] * **Singapore National Stroke Association (SNSA):** [[https://www.snsa.org.sg/](https://www.snsa.org.sg/)] * **S3 (Singapore Stroke Support Station):** [[http://www.s3.org.sg/](http://www.s3.org.sg/)]  I wish you all the best in your recovery journey. |
| **5** | How do I adjust my routines after my stroke without making things worse? Can I still do daily tasks with my stroke hand, and is it possible to resume activities like driving and going to work? | I'm not a medical professional, but I can offer some general guidance. It's crucial to consult with your healthcare team for personalized advice tailored to your specific situation.  After a stroke, adjusting routines and gradually reintroducing daily tasks is an important part of rehabilitation. Here are some general tips:  1. **Consult with Your Healthcare Team:**  - Work closely with your healthcare professionals, including physiotherapists and occupational therapists. They can assess your abilities and provide personalized guidance on what activities are safe and beneficial for you.  2. **Gradual Progression:**  - Take a gradual approach to rebuilding your routines. Start with simple tasks and gradually increase complexity as you gain strength and confidence.  3. **Use of Stroke Hand:**  - Depending on the severity of the stroke, you may be able to use your affected hand for daily tasks with practice and rehabilitation. Your healthcare team can provide exercises to improve hand function.  4. **Adaptive Techniques:**  - Explore adaptive techniques and tools to make daily tasks easier. For example, there are assistive devices that can help with gripping objects or tools designed for one-handed use.  5. **Driving and Work:**  - The ability to drive and return to work will depend on the extent of your recovery and the specific requirements of these activities. Consult with your healthcare team, especially if you've experienced any physical or cognitive changes that may impact your ability to drive safely.  6. **Rehabilitation Programs:**  - Participate in rehabilitation programs designed to improve mobility, coordination, and cognitive functions. These programs can be tailored to your needs and can significantly contribute to your recovery.  7. **Emotional Support:**  - Stroke recovery can be emotionally challenging. Seek support from friends, family, or support groups. Emotional well-being is an important aspect of the overall recovery process.  Remember, each stroke survivor's situation is unique, and what works for one person may not be applicable to another. Always follow the advice of your healthcare team, and don't hesitate to communicate any concerns or questions you may have. They can provide the most accurate guidance based on your specific condition and needs. | I understand your concerns about adjusting your routines after a stroke. While I cannot provide medical advice, I can offer some general information and resources that may help you navigate this process safely and effectively.  **Adjusting Routines:**  * **Consult your therapist:** This is crucial! Your therapist can assess your specific limitations and capabilities and create a personalized plan to gradually and safely adjust your routines. They can also guide you on safe ways to perform daily tasks and modify activities as needed. * **Start slow and gradually increase intensity:** Don't push yourself too hard initially. Begin with small, manageable tasks and gradually increase the complexity and duration as you regain strength and coordination. * **Listen to your body:** Pay close attention to any pain, discomfort, or fatigue. If you experience any of these, stop the activity and rest. Pushing too hard can hinder your recovery and even worsen your condition. * **Focus on functional tasks:** Prioritize activities that are essential for daily living, such as self-care, dressing, and cooking. This will help you regain independence and improve your quality of life.  **Using Your Stroke Hand:**  * **Rehabilitation is key:** Work with your therapist to develop exercises and techniques to improve dexterity and strength in your stroke hand. This may involve using assistive devices or practicing specific hand movements. * **Adapt and modify tasks:** Look for ways to adapt everyday tasks to accommodate your stroke hand. For example, use larger utensils, grip aids, or voice-activated devices. * **Explore adaptive equipment:** There's a wide range of adaptive equipment available to help you perform daily tasks with your stroke hand. Talk to your therapist about options that may be suitable for you.  **Resuming Activities:**  * **Driving:** Your ability to drive after a stroke depends on several factors, including the severity of your stroke, your cognitive abilities, and your physical limitations. You'll need to undergo a driving evaluation by a licensed occupational therapist or driving rehabilitation specialist. * **Returning to work:** Discuss your return to work with your doctor, therapist, and employer. They can help you create a plan for a safe and successful transition back to work. This may involve adjusted hours, modified duties, or assistive technology.  **Remember:**  * Recovery from stroke is a unique and individual process. Be patient with yourself and celebrate your progress, no matter how small. * Communication is key. Talk to your healthcare team about your concerns and goals. They are there to support you on your journey.  **Additional Resources:**  * **Singapore National Stroke Association (SNSA):** [http://www.snsa.org.sg/](http://www.snsa.org.sg/): [http://www.snsa.org.sg/](http://www.snsa.org.sg/) * **S3 - Singapore Stroke Support Station:** [http://www.s3.org.sg/](http://www.s3.org.sg/): [http://www.s3.org.sg/](http://www.s3.org.sg/) * **American Stroke Association:** [https://www.strokeassociation.org/](https://www.strokeassociation.org/): [https://www.strokeassociation.org/](https://www.strokeassociation.org/)  I hope this information helps! Please remember to consult your healthcare team for personalized advice and guidance on your specific situation. |
| **6** | How can I find support for low mood after my stroke, and is this available online? What activities are helpful when I am feeling down, and will I regain my emotional control?" | I'm not a mental health professional, but I can offer some general suggestions. It's important to consult with your healthcare team for personalized advice. Here are some ways to find support for low mood after a stroke:  1. **Speak to Healthcare Professionals:** Discuss your feelings with your healthcare team, including your doctor, neurologist, or a mental health professional. They can provide guidance and may recommend therapy or counseling.  2. **Join Support Groups:** Connect with others who have experienced strokes by joining local or online support groups. Organizations such as the Stroke Support Station in Singapore may offer resources and support.  3. **Online Resources:** Explore online platforms that provide information and support for stroke survivors. Websites, forums, and social media groups can connect you with people who share similar experiences.  4. **Therapy and Counseling:** Consider individual or group therapy sessions. Cognitive-behavioral therapy (CBT) or other therapeutic approaches can be effective in managing mood and emotional challenges.  5. **Engage in Hobbies and Activities:** Pursue activities you enjoy or discover new hobbies. Engaging in activities can provide a sense of accomplishment and joy.  6. **Physical Exercise:** Regular physical activity is not only beneficial for physical rehabilitation but can also have positive effects on mood and mental well-being.  7. **Mindfulness and Relaxation Techniques:** Practice mindfulness or relaxation techniques, such as deep breathing exercises or meditation, to help manage stress and improve emotional control.  Regarding regaining emotional control, it's important to note that emotional recovery can vary among individuals. Working with healthcare professionals and following a comprehensive rehabilitation plan can contribute to emotional well-being over time.  Remember, it's crucial to communicate openly with your healthcare team about your feelings and experiences so they can provide appropriate support and guidance based on your specific situation. | It's understandable to experience low mood after a stroke, given the physical and emotional challenges it presents. Finding support during this time is crucial, and both online and offline resources exist to help you.  **Seeking Support:**  **Offline:**  * **Your Doctor:** Talk openly about your low mood with your doctor. They can assess your situation, offer guidance, and potentially recommend medication or therapy if needed. * **Stroke Support Groups:** Connecting with other stroke survivors who understand your challenges can be incredibly valuable. Look for groups in your area through the Singapore National Stroke Association (SNSA) or Step3 Stroke Support Station. * **Mental Health professionals:** Consider seeking therapy from a psychologist or counselor specializing in post-stroke issues. They can provide individual support and develop coping mechanisms for emotional challenges.  **Online:**  * **SNSA website:** Offers resources and information on managing emotional challenges after stroke, including support groups and contact details for mental health professionals. * **Step3 website:** Provides various resources for stroke survivors, including online support groups and forums. * **National Stroke Association (US):** While based in the US, their website offers informative resources on post-stroke emotional well-being that can be helpful. * **Stroke Foundation - Australia:** Similar to the National Stroke Association, offers downloadable resources on managing mood after stroke.  **Activities for Improving Mood:**  * **Physical Activity:** Even moderate exercise releases endorphins, which elevate mood and combat stress. Engage in activities you enjoy, like walking, swimming, or dancing, as tolerated. * **Mindfulness and relaxation techniques:** Practices like meditation, deep breathing, and yoga can help manage stress and improve emotional well-being.  * **Creative activities:** Engaging in hobbies you enjoy, like painting, writing, or music, can provide a sense of accomplishment and distract from negative thoughts. * **Connecting with loved ones:** Spending time with supportive friends and family can boost mood and provide emotional support.  **Regaining Emotional Control:**  Recovering from a stroke is a journey, and regaining emotional control takes time and consistent effort. Remember:  * **Be patient:** Don't expect immediate improvement. Healing is gradual, and there will be good and bad days. * **Practice self-compassion:** Be kind to yourself and acknowledge your challenges. Celebrate small victories and focus on progress, not perfection. * **Seek professional help when needed:** Don't hesitate to reach out to mental health professionals if you need additional support managing your emotional state.  Remember, you're not alone in this. With the right support and commitment, you can navigate this challenging phase and improve your emotional well-being after your stroke. |
| **7** | How do I connect with others facing similar challenges locally and who can I talk to for psychological support? Also, where can I get financial assistance, and will there be ongoing support after rehab? | Connecting with others facing similar challenges, seeking psychological support, and finding financial assistance are important aspects of stroke recovery. Here are some suggestions specific to Singapore:  1. **Support Groups:**  - Check with local hospitals, rehabilitation centers, or community centers for stroke support groups. These groups often provide a platform for individuals with similar experiences to share advice, challenges, and encouragement.  2. **Healthcare Professionals:**  - Your healthcare team, including doctors, therapists, and social workers, can offer guidance on local resources for psychological support and assistance programs. They may be able to refer you to appropriate services.  3. **National Stroke Association of Singapore (NSAS):**  - The NSAS is an organization dedicated to stroke awareness and support in Singapore. They may provide information on support groups, rehabilitation services, and other resources. Visit their website or contact them directly for assistance.  4. **Mental Health Services:**  - Reach out to mental health professionals, such as psychologists or counselors, who specialize in stroke recovery or rehabilitation. Your healthcare provider can recommend suitable practitioners.  5. **Financial Assistance:**  - For financial assistance, you can inquire with government agencies or non-profit organizations in Singapore that focus on supporting individuals with health-related challenges. Social workers at hospitals or community centers can guide you on available financial aid programs.  6. **Community Centers:**  - Local community centers often organize activities and programs for residents, including those focused on health and well-being. Check with your nearest community center for information on relevant support groups or events.  7. **Post-Rehabilitation Support:**  - Speak with your healthcare team about post-rehabilitation support. They can provide information on continued therapy, follow-up care, and ongoing resources for your recovery journey.  Remember, it's crucial to involve your healthcare professionals in these discussions to ensure you receive accurate and personalized guidance. They can connect you with the appropriate resources based on your individual needs and circumstances. | I understand that dealing with the aftermath of a stroke can be overwhelming, and you're looking for support on multiple fronts. Here's what I can share to help you connect with others, access psychological support, explore financial aid options, and understand ongoing support after rehab:  **Connecting with Others:**  * **Support Groups:**  * **SNSA Stroke Support Groups:** The Singapore National Stroke Association (SNSA) offers stroke support groups for survivors and caregivers. These groups provide a safe space to share experiences, gain encouragement, and learn from others facing similar challenges. You can find a list of groups and contact information here: <invalid URL removed>: <invalid URL removed>  * **S3 Stroke Support Station:** S3 also offers stroke support groups and activities that can help you connect with others. Visit their website for more details: [https://www.s3.org.sg/programmes](https://www.s3.org.sg/programmes): [https://www.s3.org.sg/programmes](https://www.s3.org.sg/programmes)  * **Online Communities:** Consider joining online forums or communities for stroke survivors. These platforms can connect you with people from all over the world who understand what you're going through. Some popular options include:  * Stroke Association Support Forum: <invalid URL removed>: <invalid URL removed>  * My Stroke Guide: <invalid URL removed>: <invalid URL removed>  **Psychological Support:**  * **SNSA Counselling Services:** SNSA offers professional counselling services to help you cope with the emotional challenges of stroke. You can find more information here: <invalid URL removed>: <invalid URL removed> * **S3 Caregiver Support Programme:** S3 provides support groups and workshops specifically for caregivers of stroke survivors. This can be a valuable resource if you're caring for someone who has had a stroke. Learn more here: [https://www.s3.org.sg/caregivers-programme](https://www.s3.org.sg/caregivers-programme): [https://www.s3.org.sg/caregivers-programme](https://www.s3.org.sg/caregivers-programme) * **National Council of Social Service (NCSS):** NCSS provides a directory of social service agencies that offer counselling and mental health support. You can search their directory based on your specific needs and location: <invalid URL removed>: <invalid URL removed>  **Financial Assistance:**  * **S3 Financial Assistance Scheme:** S3 offers a financial assistance scheme to help stroke survivors who face financial difficulties accessing rehabilitation services. Learn more and check your eligibility here: <invalid URL removed>: <invalid URL removed> * **Patient Assistance Funds:** Some hospitals and medical institutions in Singapore offer patient assistance funds to help patients who cannot afford medical care. Contact your hospital or healthcare provider to inquire about their specific programs. * **Edusave for Medical Needs (EMAS):** This government scheme helps Singaporeans and Permanent Residents with Medisave balances pay for certain medical and healthcare expenses, including stroke rehabilitation. Find more information here: <invalid URL removed>: <invalid URL removed>  **Ongoing Support after Rehab:**  * **Rehabilitation ** After completing your formal rehabilitation program, you may still need ongoing support to maintain your progress and manage your condition. Talk to your doctor or therapist about developing a follow-up plan that meets your individual needs. * **Support Groups and Communities:** Staying connected with support groups and online communities can provide ongoing encouragement and resources as you adjust to life after stroke.  Remember, you're not alone in this journey. There are many resources available to help you connect with others, access support, and manage the challenges of stroke recovery. |
| **8** | How can my caregiver get support and training to understand my mood changes? Also, how can he/she help me to stay active and motivated? | Support and training for caregivers can be crucial in understanding and managing mood changes after a stroke. Here are some suggestions:  1. **Stroke Support Groups:**  Encourage your caregiver to join local stroke support groups. These groups often provide a platform for caregivers to share experiences, gain insights, and learn coping strategies from others in similar situations.  2. **Caregiver Training Programs:**  Look for caregiver training programs or workshops provided by hospitals, rehabilitation centers, or community organizations. These programs often cover various aspects of stroke recovery, including understanding mood changes and providing effective support.  3. **Professional Counseling:**  A caregiver may benefit from individual or group counseling sessions with a mental health professional. This can offer emotional support, coping strategies, and a better understanding of the challenges associated with caregiving.  4. **Online Resources:**  There are numerous online resources, forums, and educational materials specifically designed for stroke caregivers. Websites of reputable stroke organizations, such as the National Stroke Association or local Singaporean stroke support organizations, can be valuable sources of information.  To help you stay active and motivated, your caregiver can consider the following strategies:  1. **Set Realistic Goals:**  Work with your healthcare team to establish achievable rehabilitation goals. Breaking down larger goals into smaller, manageable tasks can make the process more motivating.  2. **Variety in Activities:**  Include a variety of activities in your rehabilitation routine to keep things interesting. This could include physical exercises, cognitive tasks, and recreational activities tailored to your abilities and interests.  3. **Positive Reinforcement:**  Celebrate achievements, no matter how small. Positive reinforcement can boost motivation and provide a sense of accomplishment.  4. **Regular Communication:**  Maintain open communication with your healthcare team about your progress and any challenges you may be facing. Adjustments to your rehabilitation plan can be made based on your feedback.  5. **Incorporate Social Support:**  Include friends and family in your activities when possible. Social support can contribute significantly to motivation and overall well-being.  Remember, communication between you, your caregiver, and your healthcare team is crucial. Regular discussions about your needs, goals, and any concerns will help ensure a supportive and effective rehabilitation process. | It's wonderful that you're thinking about your caregiver's well-being and seeking ways to support your own recovery. Here are some tips for both your caregiver and you:  **Supporting your caregiver:**  * **Open communication:** Talk openly and honestly with your caregiver about your mood changes. Explain how they make you feel and what triggers them. Encourage them to ask questions and express their concerns. * **Share resources:** Provide your caregiver with educational materials about stroke-related mood changes, such as:  * **Singapore National Stroke Association (SNSA):** [https://www.snsa.org.sg/](https://www.snsa.org.sg/): [https://www.snsa.org.sg/](https://www.snsa.org.sg/)  * **Stroke Foundation Australia:** [https://strokefoundation.org.au/](https://strokefoundation.org.au/): [https://strokefoundation.org.au/](https://strokefoundation.org.au/) (Has good general information applicable to Singapore too)  * **American Stroke Association:** <invalid URL removed>: <invalid URL removed> * **Connect with support groups:** Encourage your caregiver to join support groups specifically for caregivers of stroke patients. Sharing experiences and tips with others can be immensely helpful.  * **SNSA Caregiver Support Group:** <invalid URL removed>: <invalid URL removed>  * **Caregiver Alliance Singapore:** <invalid URL removed>: <invalid URL removed> * **Seek professional help:** If your caregiver feels overwhelmed or struggles to cope, encourage them to seek professional counseling or therapy.  **Staying active and motivated:**  * **Set small, achievable goals:** Together, set realistic goals for physical activity and daily tasks. Start small and gradually increase difficulty as you progress. Celebrating small wins can boost motivation. * **Focus on things you enjoy:** Incorporate activities you used to enjoy before your stroke into your rehabilitation plan. Even modified versions can be beneficial. * **Engage in social activities:** Staying connected with loved ones and participating in social activities can combat feelings of isolation and improve mood. * **Seek professional guidance:** Talk to your therapist or doctor about ways to maintain motivation and manage mood changes during your recovery. They can tailor strategies specific to your needs.  Remember, communication and collaboration are key. By working together, you and your caregiver can create a supportive environment that fosters your recovery and well-being.  **Additional resources:**  * **HealthHub:** [https://www.healthhub.sg/a-z/medical-and-care-facilities/care-after-stroke](https://www.healthhub.sg/a-z/medical-and-care-facilities/care-after-stroke): [https://www.healthhub.sg/a-z/medical-and-care-facilities/care-after-stroke](https://www.healthhub.sg/a-z/medical-and-care-facilities/care-after-stroke) * **Agency for Integrated Care (AIC):** [https://www.aic.sg/caregiving/caring-for-loved-one-with-stroke/](https://www.aic.sg/caregiving/caring-for-loved-one-with-stroke/): [https://www.aic.sg/caregiving/caring-for-loved-one-with-stroke/](https://www.aic.sg/caregiving/caring-for-loved-one-with-stroke/)  I hope this information proves helpful in your journey towards recovery! |
| **9** | What exercises can I do at home to improve my physical condition after stroke and how can I find resources for these? Are there specific activities to avoid? | I'm not a medical professional, but I can offer some general suggestions for exercises that are commonly recommended for stroke survivors. However, it's crucial to consult with your healthcare team, including a physical therapist, before starting any new exercise routine to ensure it's safe and appropriate for your specific condition.  1. **Range of Motion Exercises:**  Work on gentle range of motion exercises to improve flexibility and prevent stiffness. This can include gentle stretching of the arms, legs, and neck. Your physical therapist can provide guidance on specific movements.  2. **Strength Training:**  Focus on strengthening exercises for various muscle groups. This could involve using resistance bands, light weights, or bodyweight exercises. Start with low resistance and gradually progress under the guidance of your therapist.  3. **Balance and Coordination Exercises:**  Include activities that challenge your balance and coordination. This might involve standing on one leg, walking heel-to-toe, or practicing balance exercises recommended by your physical therapist.  4. **Cardiovascular Exercise:**  Engage in low-impact cardiovascular activities, such as walking or stationary cycling. Gradually increase the duration and intensity based on your tolerance and recommendations from your healthcare team.  5. **Functional Activities:**  Incorporate daily activities into your routine that mimic real-world movements. This could include practicing getting up from a chair, walking short distances, or reaching for items at different heights.  6. **Adaptive Yoga or Tai Chi:**  Modified yoga or tai chi exercises can help with flexibility, balance, and relaxation. Look for classes specifically designed for individuals with stroke or those with limited mobility.  To find resources for these exercises:  1. **Consult Your Healthcare Team:**  Your physical therapist or rehabilitation team can provide personalized exercises and guidance based on your specific needs and abilities.  2. **Local Stroke Support Groups:**  Connect with local stroke support groups or community centers that may offer exercise programs tailored for stroke survivors.  3. **Online Resources:**  There are many reputable online resources providing exercises for stroke survivors. Websites of stroke associations, rehabilitation organizations, or YouTube channels dedicated to stroke recovery may offer valuable guidance.  As for activities to avoid, it's crucial to consult with your healthcare team for personalized advice. Generally, activities that put excessive strain on your affected muscles or joints should be approached with caution. If you experience pain, discomfort, or worsening symptoms during any exercise, stop immediately and consult your healthcare provider.  Always prioritize safety, start with gentle movements, and progress gradually under the supervision of your healthcare team to ensure a safe and effective rehabilitation process. | While I can't give specific medical advice, I can help you explore general information about stroke rehabilitation exercises and resources in Singapore. Remember, it's **crucial to discuss any exercise plans with your healthcare team** before starting, as they can tailor them to your individual needs and limitations.  **Exercises for Stroke Rehabilitation:**  Here are some **general categories** of exercises commonly used in stroke rehabilitation:  * **Range-of-motion exercises:** These improve flexibility and joint mobility. Examples include arm circles, shoulder shrugs, and ankle rotations. * **Strengthening exercises:** These build muscle strength and endurance. Examples include bicep curls, leg lifts, and squats (modified if needed). * **Balance exercises:** These improve balance and coordination. Examples include heel-toe walking, single-leg stands, and Tai Chi. * **Functional exercises:** These practice everyday activities like sit-to-stand, reaching, and grasping.  **Finding Resources:**  Here are some resources you can use to find specific exercises and information:  * **National Stroke Association (SNSA):** [https://www.snsa.org.sg/](https://www.snsa.org.sg/): [https://www.snsa.org.sg/](https://www.snsa.org.sg/) They offer resources like exercise videos and guides. * **S3 (Stroke Support Station):** [http://www.s3.org.sg/](http://www.s3.org.sg/): [http://www.s3.org.sg/](http://www.s3.org.sg/) They provide support groups and rehabilitation resources. * **HealthHub - Stroke: Post-stroke Care:** [https://www.healthhub.sg/a-z/medical-and-care-facilities/care-after-stroke](https://www.healthhub.sg/a-z/medical-and-care-facilities/care-after-stroke): [https://www.healthhub.sg/a-z/medical-and-care-facilities/care-after-stroke](https://www.healthhub.sg/a-z/medical-and-care-facilities/care-after-stroke) This government website offers general information on post-stroke care and exercise. * **YouTube:** Search for "stroke rehabilitation exercises" to find instructional videos with various exercises.  **Activities to Avoid:**  It's crucial to **avoid exercises that cause pain, dizziness, or instability**. Also, consult your healthcare team about any activities they specifically advise against based on your condition.  Remember, consistency is key. Start slowly and gradually increase the intensity and duration of your exercises as tolerated.   I hope this information helps you explore stroke rehabilitation exercises safely and effectively. Best wishes in your recovery journey! |
| **10** | What foods should I eat or avoid after a stroke? Is there a special diet to follow, and how can I find this information? | I'm not a doctor, but I can offer some general information. After a stroke, it's essential to focus on a healthy and balanced diet to support your overall well-being and recovery. It's crucial to consult with your healthcare team for personalized advice based on your specific health condition, medications, and dietary needs. However, here are some general tips:  1. **Fruits and Vegetables:** Include a variety of colorful fruits and vegetables in your diet. They provide essential vitamins, minerals, and antioxidants.  2. **Whole Grains:** Opt for whole grains such as brown rice, quinoa, and whole wheat bread, which can provide fiber and nutrients.  3. **Lean Proteins:** Choose lean protein sources like fish, poultry, beans, and legumes. Fish rich in omega-3 fatty acids, such as salmon, can be beneficial.  4. **Healthy Fats:** Include sources of healthy fats, such as avocados, nuts, seeds, and olive oil. These fats can contribute to heart health.  5. **Limit Salt and Processed Foods:** Reduce your intake of processed foods, as they often contain high levels of sodium. Too much salt can contribute to high blood pressure.  6. **Hydration:** Stay well-hydrated by drinking plenty of water throughout the day.  It's crucial to manage other health conditions, such as diabetes and high blood pressure, which can contribute to stroke risk. Your healthcare team will likely provide guidance on managing these conditions through diet and lifestyle.  Always consult with your healthcare professionals, such as a registered dietitian or your treating physician, for personalized dietary advice tailored to your specific needs and health status. They can take into account your medical history, medications, and any other individual factors that may impact your dietary requirements. | It's important to understand that I cannot provide specific dietary advice as I am not a medical professional. However, I can offer some general information and resources to help you navigate dietary choices after a stroke in Singapore:  **General Dietary Guidelines:**  * **Focus on a balanced diet:** Prioritize fruits, vegetables, whole grains, lean protein sources like fish and poultry, and low-fat dairy products.  * **Limit unhealthy fats:** Reduce saturated and trans fats found in fried foods, processed meats, and sugary snacks. * **Minimize sodium:** High sodium intake can worsen blood pressure, so opt for low-sodium alternatives and limit added salt. * **Control sugar intake:** Excessive sugar can contribute to various health issues, so choose fruits and unsweetened beverages over sugary drinks and processed foods. * **Stay hydrated:** Drink plenty of water throughout the day to support overall health and recovery.  **Special Diets:**  * While there's no single "stroke diet," healthcare professionals may recommend specific adjustments based on your individual needs and medical conditions, such as diabetes or high blood pressure. * A Mediterranean-style diet rich in fruits, vegetables, whole grains, and healthy fats may be beneficial for post-stroke recovery. * Seek guidance from a registered dietitian or nutritionist in Singapore who can create a personalized plan aligned with your health goals and cultural preferences.  **Finding Information:**  * **HealthHub Singapore:** Their website offers resources on stroke care and diet, including a downloadable booklet: [https://www.healthhub.sg/a-z/support-groups-and-others/stroke-log-book](https://www.healthhub.sg/a-z/support-groups-and-others/stroke-log-book): [https://www.healthhub.sg/a-z/support-groups-and-others/stroke-log-book](https://www.healthhub.sg/a-z/support-groups-and-others/stroke-log-book) * **Singapore National Stroke Association (SNSA):** They provide educational materials and connect you with healthcare professionals: [https://www.snsasg.org/](https://www.snsasg.org/): [https://www.snsasg.org/](https://www.snsasg.org/) * **Dietitians Association of Singapore (DAS):** Find a registered dietitian for personalized dietary advice: [https://www.das.org.sg/](https://www.das.org.sg/): [https://www.das.org.sg/](https://www.das.org.sg/)  **Remember:**  * Consult your doctor or healthcare team before making any significant dietary changes after a stroke. * They can assess your individual needs and recommend the most suitable dietary approach for optimal recovery. * Communicate openly with your healthcare team about any concerns or difficulties you may face in managing your diet post-stroke.  I hope this information empowers you to make informed dietary choices and find the right resources for personalized support in Singapore. |
